# Identification of CircRNA–miRNA–mRNA Regulatory Network in Gastrointestinal Stromal Tumor

**DOI:** 10.3389/fgene.2020.00403

**Published:** 2020-05-28

**Authors:** Fang-wen Zou, Ding Cao, Yi-fang Tang, Long Shu, Zhongkun Zuo, Lei-yi Zhang

**Affiliations:** ^1^Department of Oncology, The Second Xiangya Hospital of Central South University, Changsha, China; ^2^Department of Minimally Invasive Surgery, The Second Xiangya Hospital of Central South University, Changsha, China; ^3^Department of Anesthesiology, The Second Xiangya Hospital of Central South University, Changsha, China

**Keywords:** gastrointestinal stromal tumors, circular RNA, miRNA, mRNA, biomarker

## Abstract

Circular RNA (circRNA) abnormal expression and regulation are involved in the occurrence and development of a variety of tumors. However, the role of circRNAs still remains unknown in gastrointestinal stromal tumors (GISTs). In the present study, the differential circRNA expression profile of GISTs was screened by human circRNAs chip and verified by qRT-PCR. The circRNA–miRNA–mRNA regulatory network was constructed using the cytoHubba plugin based on the Cytoscape software. Gene ontology (GO) and Kyoto Encyclopedia of Genes and Genomes (KEGG) analyses were performed to explore circRNA functions. Six significantly differential circRNAs were also verified in 20 pairs of GISTs and adjacent tissues by qRT-PCR. The result showed that a total of 543 differentially expressed circRNAs were identified in GISTs, of which 242 were up-regulated and 301 were down-regulated. Additionally, the circRNA–miRNA–mRNA network contained six circRNAs, 30 miRNAs, and 308 mRNAs, and the targeted mRNAs were associated with “regulation of biological process,” “intracellular organelle,” “protein binding,” and enriched in Wnt signaling pathway. Furthermore, qRT-PCR demonstrated that hsa_circRNA_061346, hsa_circRNA_103114, and hsa_circRNA_103870 were significantly up-regulated in GISTs (*n* = 20), and hsa_ circRNA_405324, hsa_circRNA_406821, and hsa_circRNA_000361 were dramatically down-regulated in GISTs (*n* = 20). In addition, all of these circRNAs were shown to have high diagnostic values, and most of them were significantly associated with tumor size, mitotic figure, and malignant degrees in GISTs (*P* < 0.05). Therefore, we concluded that circRNAs were abnormally expressed in GISTs, and the circRNA–miRNA–mRNA regulatory network plays an important role in the occurrence and development of GISTs. Also, the identified six candidate circRNAs might be critical circRNAs and may present as potential diagnostic biomarkers for GISTs.

## Introduction

Gastrointestinal stromal tumors are rarely one of the gastrointestinal carcinomas that originate from mesenchymal tissue. GISTs are characterized by expression of CD117 receptor in cells and have variable biological phenotypes ranging from benign to highly malignant (Gautam, 2020). As one of the most common non-epithelial neoplasms, they are mainly located in the stomach (55.6%) and small intestine (31.8%) (Tao et al., 2020). Radical surgery is the preferred treatment, and molecular target therapy, such as imatinib, can improve the survival of advanced patients with c-kit and/or PDGFRα mutations (Gupta and Rateria, 2020). However, a few effective tumor biomarkers are used for GIST diagnosis and prediction (Etherington and DeMatteo, 2019).

Circular RNA is a novel class of endogenous non-coding RNA characterized with 3′- and 5′-ends covalently linked in a closed-loop structure (Mahmoudi et al., 2020), which makes circRNAs resistant to exonucleases and more stable than traditional linear RNA, such as lncRNA and miRNA (Ding et al., 2020). Accordingly, the circRNAs can be divided into four types according to the source (Meng et al., 2017): exonic circRNAs (ecircRNA), intronic circRNA (ciRNA), exonic–intronic circRNA (EIciRNA), and intergenic circRNAs. Among them, 80% of circRNAs are ecircRNA. circRNAs may act as microRNA (miRNA/miR) sponges by competitively binding to miRNA response elements to influence downstream target gene expression, as well as affecting gene function at a post-translational level, and the same circRNA can regulate multiple miRNAs. Also, the same miRNAs can regulate multiple mRNA genes, thereby forming a large circRNA–miRNA–mRNA competitive network to affect the development of tumors (Xu S. et al., 2018). Also, some circRNAs can exert their activities via interaction with some proteins. Even then, some ecircRNAs may participate in the assembly and protein ribosomes translation. It was reported that circRNA is involved in various biological processes, including signal transduction and transcription, cell cycle regulation, RNA-binding protein, responses to stress, protein metabolism, cellular immunity, and cell structure (Jiang et al., 2018). Recent studies (Lu et al., 2018; Chaichian et al., 2020) have also demonstrated that circRNA abnormal expression and regulation are involved in the occurrence and development of a variety of tumors. Therefore, circRNAs are of great importance as a biomarker for cancer diagnosis, cancer prediction, and treatment feedback, and may even serve as targets for cancer treatment.

In this study, we first analyzed the circRNA differential expression profile in GISTs using circRNA chip and identified six potential key circRNAs by qRT-PCR. Also, the circRNA–miRNA–mRNA network was constructed, and the GO and KEGG pathway were performed via bioinformatics analysis. Our study provides a novel insight into the molecular mechanisms of GISTs from the circRNA–miRNA–mRNA network view, and these circRNAs gave new direction for diagnosis and treatment of GISTs.

## Materials and Methods

### Patients and Samples

Twenty pairs of GISTs and adjacent tissues were collected from The Second Xiangya Hospital of Central South University. All pathological specimens were experienced pathologists confirmed, did not accept the pre-operative radiotherapy, chemotherapy, and imatinib targeted therapy. The clinicopathological features are shown in Table 1. All tissues were collected during surgical operation and instantly stored in liquid nitrogen. The present project was permitted by the ethics committee of The Second Xiangya Hospital of Central South University, and informed consents were obtained from all the participants.

**TABLE 1:** The clinicopathological features of GISTs patients.

**Variables**	**Cases (*n*)**
Total	20
**Age (years)**	
≥60	12
<60	8
**Gender**	
Male	15
Female	5
**Tumor size (cm)**	
≤5	9
>5	11
**Mitotic figure (HPF)**	
≤5/50	13
>5/50	7
**Malignant degrees**	
Low/Moderate risk	12
High risk	8

### CircRNA Chip Detection

Total RNAs were extracted by RNeasy Mini Kit (Qiagen, Hilden, Germany). Total RNA from each sample was quantified using the NanoDrop ND-1000. The sample preparation and microarray hybridization were performed based on the Arraystar’s standard protocols. Total RNAs were digested with Rnase R to remove linear RNAs and enrich CircRNAs. Then, the enriched CircRNAs were amplified and transcribed into fluorescent cRNA (Arraystar Super RNA Labeling Kit; Arraystar). The labeled cRNAs were hybridized onto the Arraystar Human circRNA Array V2 (8 × 15 K; Arraystar). Agilent Feature Extraction software (version 11.0.1.1) was used to analyze acquired array images. Quantile normalization and subsequent data processing were performed using the R software limma package. Differentially expressed circRNAs with statistical significance between two groups were identified through volcano plot filtering. Differentially expressed circRNAs between two samples were identified through fold change filtering. Hierarchical clustering was performed to show the distinguishable circRNA expression pattern among samples.

### Quantitative Real-Time PCR

Total RNAs were extracted using RNeasy Mini kit (Qiagen, Hilden, Germany). Then, RNA was reversed into complementary DNA (cDNA) by SuperScript III Reverse Transcriptase (Invitrogen). qRT-PCR was performed with 95.0°C for 3 min, and 39 circles of 95.0°C for 10 s and 60°C for 30 s using SYBR Green PCR Master Mix system. The relative expression levels were calculated using the 22^−ΔΔ*Ct*^ method. RNA levels were normalized to GAPDH expression. The forward (F) and reverse (R) primer sequences for qRT-PCR were designed and synthesized by Shanghai Kangcheng Co., Ltd. (Chinese).

### CircRNA–miRNA–mRNA Interaction Prediction

The fundamental structure of circRNAs was predicted using Cancer-Specific circRNA (CSCD^1^). circRNA–miRNA interactions were predicted using TargetScan and miRanda databases, and miRNA target gene was predicted using TargetScan, miRanda v5, and miBase prediction databases. Candidate miRNAs and mRNAs should be overlapped in at least two databases. Arraystar’s miRNA target prediction software site: miRanda v5^2^, TargetScan^3^, and mibase^4^. The circRNA–miRNA–mRNA competitive network (cirCeNET) was visualized by Cytoscape software (version 3.6.1^5^).

### Gene Ontology (GO) and KEGG Pathway Analysis

Gene ontology and KEGG pathways analysis was used to determine the function of candidate mRNAs in circRNA–miRNA–mRNA competitive network. DAVID^6^ was used to predict the enriched functional categories and enriched signaling pathways. GO term, including BP, CC, MF, and KEGG pathway with *P* < 0.05 and FDR < 0.05 were considered as statistically significant.

### Statistical Analysis

All data were analyzed by SPSS17.0 statistics software. Paired *t*-test was employed for the comparison of two groups. Chi-square test was used to investigate the relationship between circRNA expression and clinicopathologic features of GISTs patients. *P* < 0.05 was considered as statistically significant.

## Results

### Differential CicrRNA Expression Profiles Were Established Successfully

**FIGURE 1:**
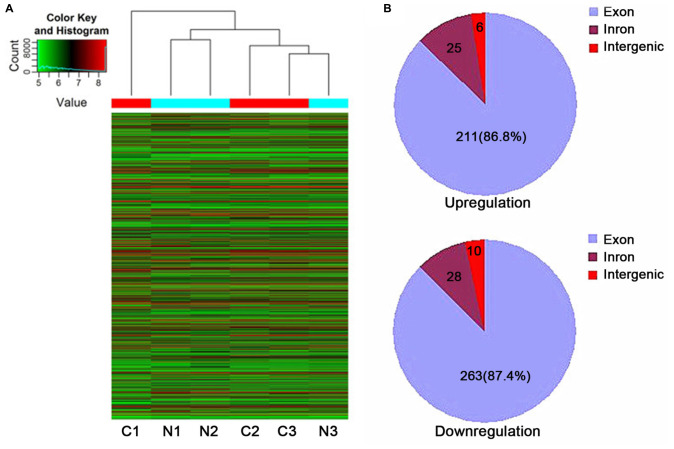
Characterization of circRNA expression in GISTs. **(A)** Hierarchical cluster map. **(B)** The origin of upregulated and downregulated circRNAs. N, adjacent normal tissues; C, GIST tissues.

The box plot showed similar distributions of tissues. In the volcano plots, differentially expressed circRNAs were categorized using fold change and *P* values. The scatter plots demonstrated the variation of differentially expressed circRNAs. Hierarchical cluster analysis showed differentially expressed circRNAs in GISTs with fold change >1.5 and *P* < 0.05 (Figure 1A). After normalization and data analysis, compared with adjacent tissues, a total of 543 differentially expressed circRNAs were identified, including 242 up-regulated circRNAs and 301 down-regulated circRNAs, of which, exonic circRNAs accounted for 86.8% in up-regulated circRNAs and 87.4% in down-regulated circRNAs (Figure 1B). The top 20 significantly up- and down-regulated circRNAs are listed in Tables 2 and 3.

**TABLE 2:** Top 20 significantly up-regulated circRNAs.

**circRNA**	***P*-value**	**FDR**	**FC (abs)**	**Regulation**	**source**	**chrom**	**strand**	**type**	**GeneSymbol**
hsa_circRNA_061346	0.049017552	0.541205763	6.9877315	up	circBase	chr21	−	exonic	APP
hsa_circRNA_103114	0.027813714	0.541205763	6.8848564	up	circBase	chr21	−	exonic	APP
hsa_circRNA_103223	0.040961154	0.541205763	6.5339969	up	circBase	chr22	−	exonic	DDX17
hsa_circRNA_100582	0.02164493	0.541205763	4.5762691	up	circBase	chr10	+	exonic	ZEB1
hsa_circRNA_001481	0.047719242	0.541205763	4.5058956	up	circBase	chr5	−	sense overlapping	EMB
hsa_circRNA_103128	0.03858124	0.541205763	4.1057473	up	circBase	chr21	+	exonic	DYRK1A
hsa_circRNA_103870	0.039001555	0.541205763	4.0573338	up	circBase	chr5	+	exonic	SMA4
hsa_circRNA_404643	0.041732555	0.541205763	4.0082859	up	25070500	chr1	−	exonic	PIK3C2B
hsa_circRNA_004182	0.039090826	0.541205763	3.428553	up	circBase	chr2	+	intronic	CRIM1
hsa_circRNA_103977	0.03760255	0.541205763	3.426974	up	circBase	chr5	+	exonic	ARHGAP26
hsa_circRNA_104076	0.047784401	0.541205763	3.2920026	up	circBase	chr6	−	exonic	KIF13A
hsa_circRNA_101504	0.043980697	0.541205763	3.215775	up	circBase	chr15	+	exonic	PDIA3
hsa_circRNA_102510	0.029244589	0.541205763	3.1147657	up	circBase	chr19	+	exonic	LSM14A
hsa_circRNA_002164	0.048545781	0.541205763	3.082576	up	circBase	chr18	−	exonic	SS18
hsa_circRNA_103224	0.006590126	0.541205763	3.0780956	up	circBase	chr22	−	exonic	DDX17
hsa_circRNA_100915	0.030601002	0.541205763	3.0777302	up	circBase	chr11	−	exonic	PICALM
hsa_circRNA_403471	0.003668259	0.541205763	3.060178	up	25242744	chr5	+	exonic	ARHGAP26
hsa_circRNA_102248	0.047545686	0.541205763	3.056733	up	circBase	chr17	+	exonic	TBCD
hsa_circRNA_404446	0.005776341	0.541205763	3.0361211	up	25070500	chr1	−	exonic	CAPZB
hsa_circRNA_102378	0.045317666	0.541205763	2.9997955	up	circBase	chr18	+	exonic	ZNF532

**TABLE 3:** Top 20 significantly down-regulated circRNAs.

**circRNA**	***P*-value**	**FDR**	**FC (abs)**	**Regulation**	**source**	**chrom**	**strand**	**type**	**GeneSymbol**
hsa_circRNA_405324	0.013412911	0.541205763	4.3231649	down	25070500	chr15	+	sense overlapping	STARD9
hsa_circRNA_405443	0.028911656	0.541205763	3.3381633	down	25070500	chr16	+	intronic	NDE1
hsa_circRNA_406309	0.01797211	0.541205763	3.1653952	down	25070500	chr3	+	intronic	CMSS1
hsa_circRNA_100075	0.029545308	0.541205763	3.1382274	down	circBase	chr1	−	exonic	EMC1
hsa_circRNA_406821	0.020949363	0.541205763	3.0380172	down	25070500	chr6	+	exonic	ARMC2
hsa_circRNA_000361	0.027240076	0.541205763	3.0118108	down	circBase	chr3	−	antisense	PLCL2
hsa_circRNA_082335	0.035559522	0.541205763	3.008493	down	circBase	chr7	+	exonic	KLHDC10
hsa_circRNA_102207	0.013654637	0.541205763	3.0013356	down	circBase	chr17	+	exonic	AFMID
hsa_circRNA_405825	0.015220854	0.541205763	2.522762	down	25070500	chr2	+	exonic	KLF11
hsa_circRNA_074660	0.017052344	0.541205763	2.4763773	down	circBase	chr5	−	exonic	ATOX1
hsa_circRNA_104924	0.01941688	0.541205763	2.4549169	down	circBase	chr9	+	exonic	MVB12B
hsa_circRNA_056037	0.027008503	0.541205763	2.4478589	down	circBase	chr2	−	exonic	BUB1
hsa_circRNA_406780	0.028354789	0.541205763	2.3707217	down	25070500	chr6	−	sense overlapping	DNPH1
hsa_circRNA_024371	0.025742814	0.541205763	2.36132	down	circBase	chr11	+	exonic	PAFAH1B2
hsa_circRNA_061284	0.026417014	0.541205763	2.30245	down	circBase	chr21	+	exonic	USP25
hsa_circRNA_100456	0.009103973	0.541205763	2.2394652	down	circBase	chr1	+	exonic	KCNK2
hsa_circRNA_083919	0.037676327	0.541205763	2.213904	down	circBase	chr8	+	exonic	UNC5D
hsa_circRNA_406295	0.017374416	0.541205763	2.2027287	down	25070500	chr3	+	sense overlapping	SUCLG2-AS1
hsa_circRNA_035426	0.040284453	0.541205763	2.2016635	down	circBase	chr15	+	exonic	TCF12
hsa_circRNA_405296	0.023543643	0.541205763	2.1972163	down	25070500	chr15	+	sense overlapping	TUBGCP5

### qRT-PCR Validation

**FIGURE 2:**
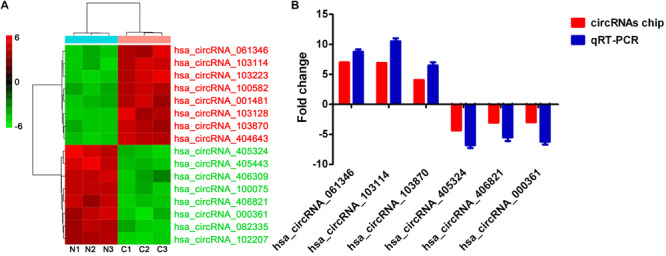
qRT-PCR validation. **(A)** The cluster heat map of the top eight most up-regulated and down-regulated circRNAs. **(B)** Comparison of Arraystar human circRNAs chip data and qRT-PCR results. circRNAs expression levels were normalized to GAPDH. N, adjacent normal tissues; C, GIST tissues.

The top eight most upregulated circRNAs with fold change >4 and *P* < 0.05 and the top eight most downregulated circRNAs with fold change >3 and *P* < 0.05 are shown in the cluster heat map (Figure 2A). qRT-PCR assay was used to assess the accuracy of circRNAs chip data. After filtering circRNAs with low raw intensity, six candidate circRNAs, including three up-regulated circRNAs (hsa_ circRNA_061346, hsa_circRNA_103114, hsa_circRNA_103870) and three down-regulated circRNAs (hsa_circRNA_405324, hsa_circRNA_406821, hsa_circRNA_ 000361) were selected for qRT-PCR analysis. The results showed that qRT-PCR results were consistent with the circRNAs chip data (Figure 2B), indicating the reliability of circRNAs chip data.

### CircRNA–miRNA–mRNA Network Construction

**FIGURE 3:**
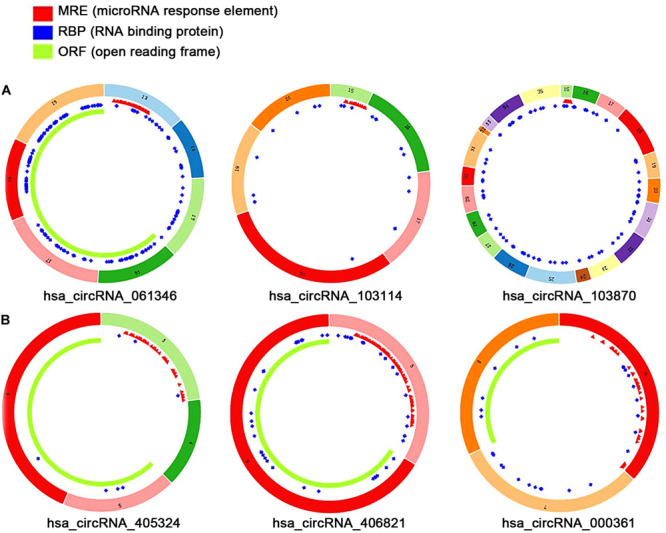
The fundamental structure modes of the six candidate circRNAs predicted by CSCD. **(A)** Up-regulated circRNAs: hsa_circRNA_061346, hsa_circRNA_103114, hsa_circRNA_103870. **(B)** Down-regulated circRNAs: hsa_circRNA_405324, hsa_ circRNA _406821, hsa_circRNA_000361.

**FIGURE 4:**
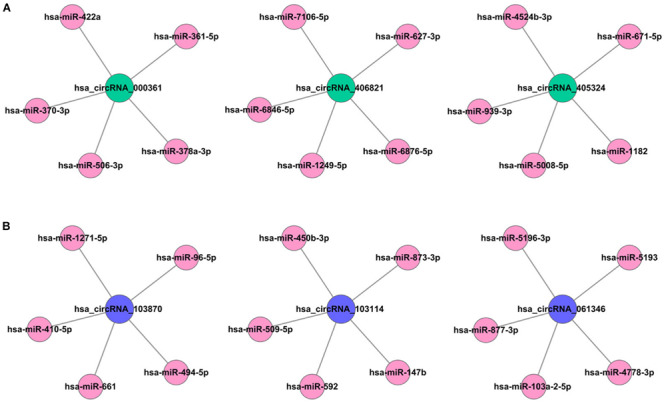
circRNA–miRNA regulatory network. **(A)** The top five targeted miRNAs of up-regulated circRNAs. **(B)** The top five targeted miRNAs of down-regulated circRNAs.

**FIGURE 5:**
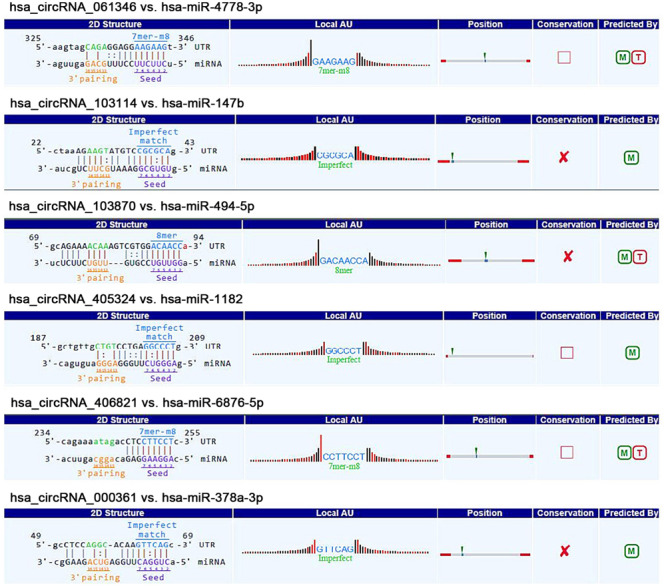
The detailed potential circRNA–miRNA interaction sites of targeted miRNAs with highest context score percentile based on TargetScan and miRanda data.

**FIGURE 6:**
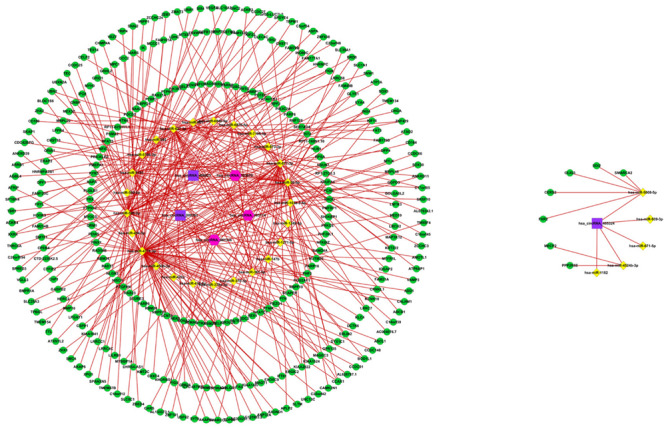
circRNA–miRNA–mRNA regulatory network. Up-regulated circRNAs, down-regulated circRNAs, miRNA, and mRNA are presented as square, hexagon, diamond, and circle, respectively.

The fundamental structure modes of the six candidate circRNAs predicted by CSCD are shown in Figure 3. To estimate the function of six candidate circRNAs, circRNA–miRNA interactions were constructed with TargetScan and miRanda databases. The top five targeted miRNAs of six candidate circRNAs are exhibited in Figure 4, and the detailed potential circRNA–miRNA interaction sites of targeted miRNAs with the highest context score percentile are shown in Figure 5. Then, the circRNA–miRNA–mRNA competitive network (cirCeNET) was visualized by Cytoscape software (version 3.6.1) based on circRNA–miRNA interactions and miRNA–mRNA interactions (Figure 6). This network contained six circRNAs, 30 miRNAs, and 308 mRNAs, which provided a comprehensive perspective into the links between circRNA, miRNA, and mRNAs in GISTs.

### GO and KEGG Pathway Analysis

**FIGURE 7:**
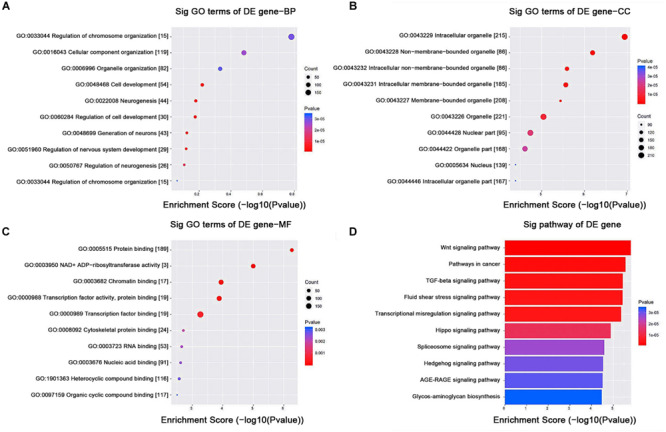
GO and KEGG pathway analysis. **(A–C)** GO annotation of targeted mRNAs with the top 10 enrichment scores for biological process, cellular component, and molecular function, respectively. **(D)** The top 10 enriched KEGG pathways. GO, gene ontology; KEGG, Kyoto Encyclopedia of Genes and Genomes.

The GO analysis demonstrated that the term with the highest enrichment score was regulation of biological process (GO:0050789) for biological process terms (BP), intracellular organelle (GO:0043229) for cellular component terms (CC), and protein binding (GO:0005515) for molecular function terms (MF), respectively. The top 10 enrichment scores are shown in Figures 7A–C. The KEGG pathway with the highest enrichment score was the Wnt signaling pathway. The top 10 enriched KEGG pathways are shown in Figure 7D.

### qRT-PCR Validation

**FIGURE 8:**
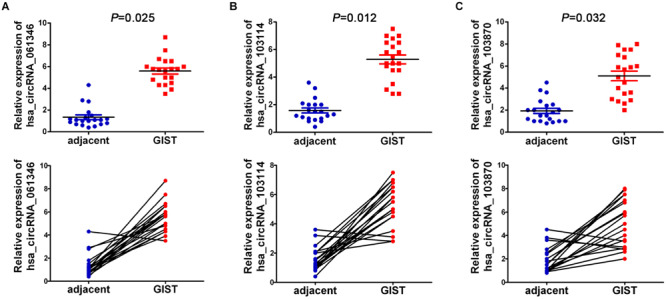
The relative expression of up-regulated circRNAs of 20 GIST tissues were detected by qRT-PCR, **(A)** hsa_circRNA_061346; **(B)** hsa_circRNA_103114; **(C)** hsa_ circRNA_103870. circRNA expression levels were normalized to GAPDH.

**FIGURE 9:**
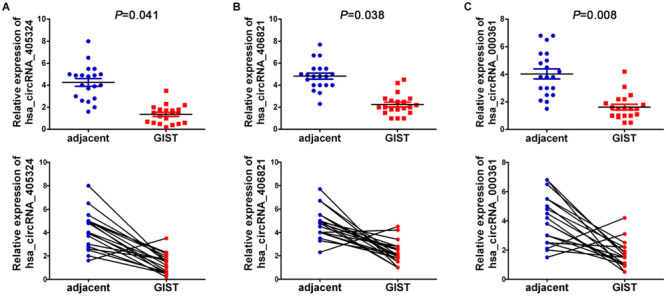
The relative expression of down-regulated circRNAs of 20 GIST tissues was detected by qRT-PCR, **(A)** hsa_circRNA_405324; **(B)** hsa_circRNA _406821; **(C)** hsa_circRNA_000361. circRNA expression levels were normalized to GAPDH.

Six significantly differential circRNAs were also verified in 20 pairs of GISTs and adjacent tissues by qRT-PCR. The results showed that circRNA_061346, circRNA_103114, and circRNA_103870 were significantly up-regulated in GIST tissues (Figure 8) (*P* < 0.05), and circRNA_405324, circRNA_ 406821, and circRNA_000361 were dramatically down-regulated in GIST tissues (Figure 9) *P* < 0.05), compared with corresponding adjacent tissues.

### Diagnosis Values of CircRNA

**FIGURE 10:**
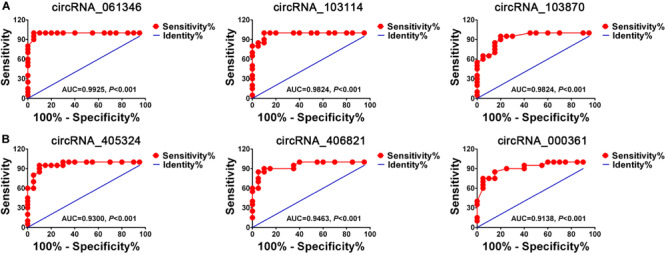
The diagnostic value of six candidate circRNAs in GISTs. **(A)** ROC curve for three up-regulated circRNAs. **(B)** ROC curve for three down- regulated circRNAs.

In order to determine the diagnostic value of six candidate circRNAs in GISTs, the ROC curve was employed. Statistical analysis demonstrated that all six candidate circRNAs had high diagnostic efficiency with AUC = 0.9925, AUC = 0.9824, AUC = 0.9231, AUC = 0.9300, AUC = 0.9463, AUC = 0.9138 for circRNA_061346, circRNA_103114, circRNA_103870 and circRNA_405324, circRNA_406821, circRNA_000361, respectively (Figure 10) (*P* < 0.05).

### Correlation of CircRNA Expressions With Clinical Pathologic Features

**FIGURE 11:**
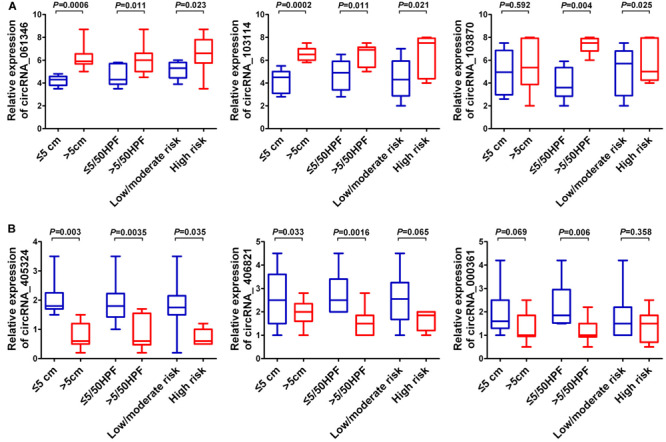
Correlation of six candidate circRNAs with clinical pathologic features. **(A)** Box plots for three up-regulated circRNAs. **(B)** Box plots for three down-regulated circRNAs. Tumor size: ≤5 cm, >5 cm; mitotic figure: ≤5/50 HPF, <5/50 HPF; malignant degrees: low risk, moderate risk, high risk.

In order to investigate the correlation of six candidate circRNA expressions with clinical–pathologic features, the median circRNA expression was used to divide the 20 pairs of GISTs tissues into higher and lower circRNA expression groups. Chi-square assay was employed for statistical analysis. The results suggested that circRNA_061346 and circRNA_103114 expressions were positively associated with tumor size, mitotic figure, malignant degrees, and circRNA_103870 expression was positively associated with tumor size, mitotic figure, but without relation to malignant degrees (Figure 11). On the contrary, circRNA_405324 expression was negatively associated with tumor size, mitotic figure, malignant degrees, and circRNA_406821 was negatively correlated with mitotic figure, malignant degrees, but not with tumor size; nevertheless, circRNA_000361 expression was only negatively related with mitotic figure (Figure 11B). However, there was no correlation with age, gender, and tumor location.

## Discussion

Gastrointestinal stromal tumors are a rare malignant tumor that occurs principally in the stomach, small intestine, and colon–rectum (Flavahan et al., 2019). K-ras gene mutation might be correlated with the mechanism of development and infiltration of GISTs, but the pathogenesis of GISTs is inadequately understood (Lasota et al., 2019). A growing body of research (Wang et al., 2018; Cai et al., 2019; Zaghlool et al., 2020) demonstrates that the development of cancer is often accompanied by abnormal expression of circRNA. Also, circRNA is characterized by inherent stability, highly conservative, and universality. Therefore, circRNA is of great importance as a biomarker for cancer screening, cancer diagnosis, cancer prediction, feedback of treatment, and prognosis. Additionally, circRNA’s abnormal expression and the circRNA–miRNA–mRNA regulatory network regulation have been increasingly demonstrated in a variety of tumors, such as circRNA_CAMK2A–miR-615-5p–fibronectin 1 network in lung adenocarcinoma metastasis (Du et al., 2019), circRNA_0006948–miR-490-3p–HMGA2 network in esophageal squamous cell carcinoma (Pan et al., 2019), circRNA_ACAP2–miR-29a/b-3p–COL5A1 network in breast cancer (Zhao et al., 2019), circRNA_51217–miRNA-646–TGFβ1/p-Smad2/3 network in prostate cancer (Xu et al., 2019), etc. At present, there are few reports about the circRNA–miRNA–mRNA regulatory network in GISTs.

In the present study, we illuminate the molecular mechanisms of circRNAs in the occurrence and development of GISTs for the first time. We first performed circRNA chip analysis to assess differential cicrRNA expression profiles in GIST tissues and corresponding non-cancer tissues. A totally of 543 differentially expressed circRNAs were identified, of which 242 were significantly upregulated and 301 were significantly downregulated in GISTs tissues. Additionally, in order to fully elucidated the function of the circRNA-related ceRNA in GISTs, six candidate circRNAs including three up-regulated circRNAs (hsa_circRNA_061346, hsa_circRNA_103114, hsa_ circRNA_103870) and three down-regulated circRNAs (hsa_circRNA_405324, hsa_ circRNA_406821, hsa_circRNA_000361) were identified to be involved in the ceRNA network. The ceRNA network consists of six circRNAs, 30 miRNAs, and 308 mRNAs. In this network, previous studies (Xu Z.H. et al., 2018; Zhang et al., 2019) show that many miRNAs, such as miR-4778-3p, miR-147b, miR-1182, and miR-378a-3p, were involved in tumor cell growth, invasion, and metastasis. Also, many targeted genes, such as ZEB1, SOX5, AKAP1, CHP1, CNBP, VEGFR, and MAGT1, play a vital function in the cell shape, movement, invasion, adhesion, and polarity formation, so as to involve in many kinds of diseases such as malignant tumors, wound healing, and so on (Rinaldi et al., 2017; Caramel et al., 2018; Hu et al., 2018).

Furthermore, 20 GIST tissues and adjacent tissues were collected to verify the expression of identified six candidate circRNAs. qRT-PCR results showed that hsa_circRNA_061346, hsa_circRNA_103114, and hsa_circRNA_103870 were significantly up-regulated in GISTs, and hsa_ circRNA_405324, hsa_circRNA _406821, hsa_circRNA_ 000361 were dramatically down-regulated in GISTs. In addition, all of these circRNAs were shown to have high diagnostic values, and most of them were significantly associated with tumor size, mitotic figure, and malignant degrees in GISTs. The six candidate circRNAs might be critical circRNAs participating in the occurrence and development of GISTs and can serve as novel potential diagnostic biomarkers for GISTs patients. Through a large literature review, very limited data are available about these circRNAs’s functions and their deregulation in cancer.

However, there are several limitations to our study. First, only 20 patients were enrolled in our study, the sample size is relatively small, and the result showed an association, rather than a definite, causal relationship. Also, the relation analysis of clinical factors and circRNAs needs to be supported by large samples. Second, in our study, we only conducted a network based on identified six critical circRNAs, miRNA, and target mRNA, but a total of 543 circRNAs were identified in GISTs. Other circRNAs may contribute as well. Third, our paper starts with a general analysis of circRNAs in GISTs, but the mechanism is not discussed in detail. Therefore, in our future work, further studies with larger groups of patients, a network based on 543 circRNAs are needed to confirm these findings, and the concrete mechanism of circRNAs in GISTs also needs to be further explored.

## Conclusion

In the present study, the differential circRNA expression profile of GISTs was established, and a total of 543 differentially expressed circRNAs were screened. In addition, the circRNA–miRNA–mRNA regulatory network was constructed. hsa_circRNA_ 061346, hsa_circRNA_103114, hsa_circRNA_103870 and hsa_circRNA_405324, hsa_ circRNA_406821, hsa_circRNA_000361 were identified as critical circRNAs in the occurrence and development of GISTs and may present as potential diagnostic biomarkers for GISTs. In brief, our study provides a new insight into the pathogenesis of GISTs from the circRNA–miRNA–mRNA regulatory network view.

## Data Availability Statement

The datasets generated for this study can be found in NCBI GEO accession GSE147303.

## Ethics Statement

The studies involving human participants were reviewed and approved by the Ethics Committee of The Second Xiangya Hospital of Central South University. The patients/participants provided their written informed consent to participate in this study.

## Author Contributions

LZ coordinated all aspects of the research. FZ and DC were responsible for the clinical sample collection. FZ participated in most molecular and cellular experiments and manuscript preparation. LS and ZZ were responsible for data analysis. All authors read and approved the final manuscript.

## Conflict of Interest

The authors declare that the research was conducted in the absence of any commercial or financial relationships that could be construed as a potential conflict of interest.

## Footnotes

1 http://gb.whu.edu.cn/CSCD

2 http://www.ebi.ac.uk/enright-srv/microcosiTi/htdo/targets/v5

3 http://www.targetscan.org

4 http://pictar.bio.nyu.edu

5 http://cytoscape.org/

6 http://www.david.abcc.ncifcrf.gov/

## Abbreviations

CircRNA: circular RNA
DAVID: Database for Annotation Visualization and Integrated Discovery
GISTs: gastrointestinal stromal tumors
GO: gene ontology
KEGG: Kyoto Encyclopedia of Genes and Genomes
MiRNAs: microRNAs
MREs: microRNA response element
ROC curve: receiver operating characteristic curve.
